# Right atrial thrombus and pulmonary thromboembolism related to ovarian hyperstimulation syndrome: A case report and literature review

**DOI:** 10.1002/ccr3.7018

**Published:** 2023-03-07

**Authors:** Amin Sayyadi, Mahsa Mahdavi, Behnam Dalfardi, Fatemeh Karami Robati, Mohsen Shafiepour

**Affiliations:** ^1^ Student Research Committee, School of Medicine Kerman University of Medical Sciences Kerman Iran; ^2^ Clinical Research Development Unit Afzalipour Hospital, Kerman University of Medical Sciences Kerman Iran; ^3^ Endocrinology and Metabolism Research Center, Institute of Basic and Clinical Physiology Sciences Kerman University of Medical Sciences Kerman Iran

**Keywords:** infertility, ovarian hyperstimulation syndrome, thrombosis, women health

## Abstract

A young lady with a history of infertility presented to the hospital with dyspnea and chest pain a few days after ovulation induction. Her manifestations were consistent with ovarian hyperstimulation syndrome (OHSS). Further investigations revealed right atrial thrombus and pulmonary thromboembolism. We successfully managed the condition with conservative therapy.

## Graphical abstract

Physicians, especially obstetricians and internists, should be aware of different possible manifestations of OHSS, and keep this differential diagnosis in mind when encountering a patient with history of ovulation induction.

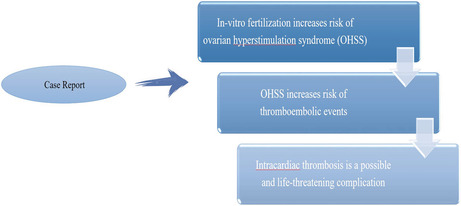

## 1 INTRODUCTION

Ovarian hyperstimulation syndrome (OHSS) is the most severe complication of in vitro fertilization (IVF), and its occurrence also increases with the rising trend of IVF.^1^, ^2^ The exact pathogenesis of this condition is unknown, but overreaction of the ovary to ovary stimulation is the main element.^1^ Young age, low body mass index, polycystic ovarian syndrome (PCOS), a rapid rise in serum estradiol, large number of growing follicles on the day of triggering, and many oocytes retrieved are the risk factors for the development of OHSS.^3^

OHSS consists of multiple components: ovarian enlargement, fluid shifts into the third space, hypovolemia, hemoconcentration, serosal effusions, ascites, renal failure, and hypercoagulable state. The last complication can result in thrombosis in multiple organs, including the lungs,^4^ brain^5^ and heart,^6^ and this can be fatal. Pregnant women have a fourfold to fivefold higher risk of thromboembolism than nonpregnant women; IVF and OHSS can increase this risk even more.^2^, ^7^, ^8^

Previous studies have mentioned cardiovascular complications of OHSS, but herein we report thrombosis in the right atrium. To the best of our knowledge, this is the first report of this complication in this location.

## 2 CASE PRESENTATION

Our patient was a 24‐year‐old lady with a history of infertility for 2 years. She was gravida two, para two with two abortions. For ovulation induction, she received Follitropin alfa (a recombinant human follicle stimulating hormone) for 2 weeks and Cetrorelix acetate (a synthetic peptide antagonist of gonadotropin‐releasing hormone) for 5 days. Two days after ovulation induction, she started complaining of mild dyspnea, the gynecologist performed an ultrasonogram (US) which showed trace fluid in the abdominopelvic cavity.

Two days after initial symptoms, she presented to the hospital with aggravation of dyspnea and sudden onset chest pain; her chest pain was retrosternal and pleuritic.

On admission, her blood pressure was 100/60 mmHg, pulse was 110 beats per minute, respiratory rate was 22 per minute, and temperature was 37°C. Peripheral arterial oxygen saturation was 85%.

Examination revealed reduced breathing sounds in the right lower lobe, positive dullness in chest percussion, and distended abdomen with positive shifting dullness.

We observed sinus tachycardia, S1Q3T3 pattern, and ST‐segment elevation in the electrocardiogram. Chest X‐ray showed right lung collapse and massive pleural effusion. With the help of the US, we drained 1300 cc of bloody pleural fluid; it was exudative; cytology, culture, and smear were all negative.

**FIGURE 1:**
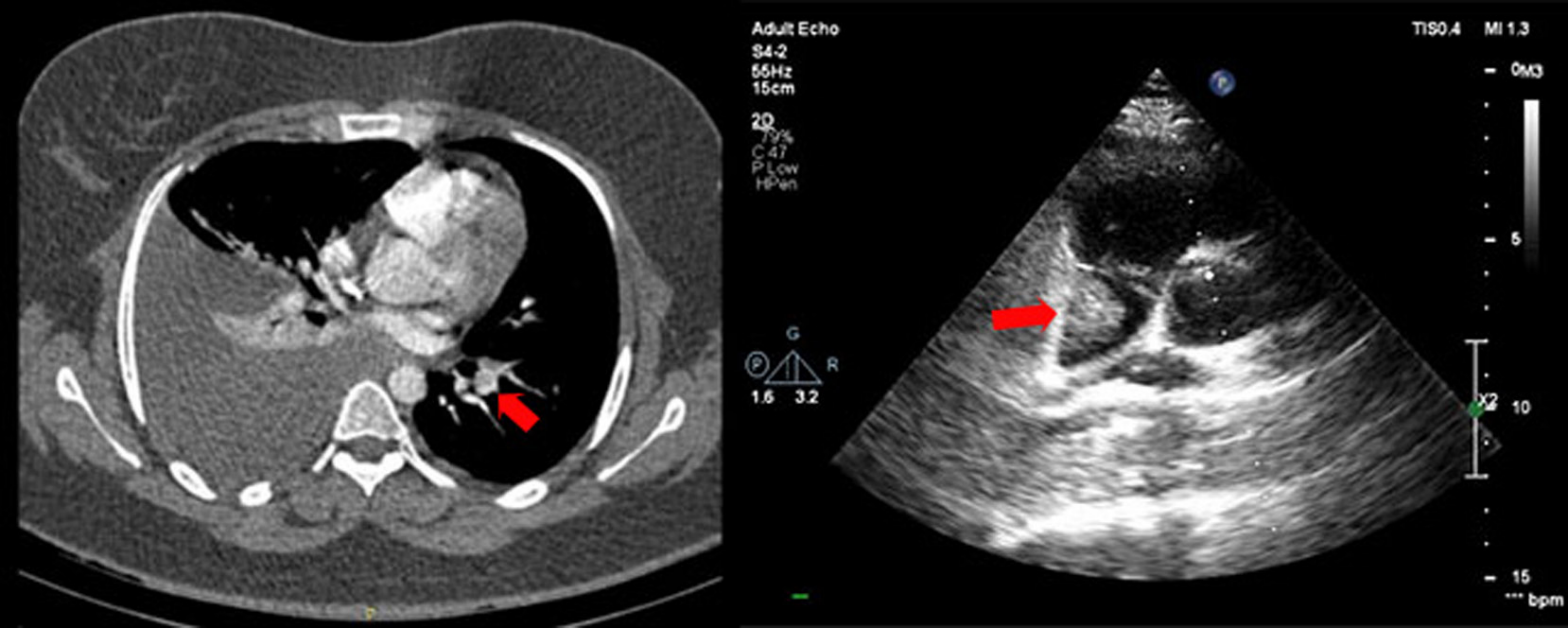
CTPA (left): The red arrow shows the clot in one of the branches of the left pulmonary artery. Echocardiography (right): The red arrow shows the large thrombus in the right atrium.

Trans‐thoracic echocardiography (TTE) showed a 1.3 × 1.7 cm mass suspicious for a clot in the right atrium and mild pericardial effusion (Figure 1, right and Video S1). Computed tomography pulmonary angiography (CTPA) revealed a filling defect on the left side (Figure 1, left). In the abdominopelvic US, we observed hemorrhagic ovarian follicles and moderate fluid in the abdominopelvic cavity. Lower extremities Doppler ultrasound showed no deep vein thrombosis (DVT).

Laboratory tests revealed high white blood cells (15.5 10^3^/μL), erythrocyte sedimentation rate (46 mm/h), and positive troponin I. Urine analysis was normal except for protein and blood being +1. Other laboratory results including hemoglobin, platelets, venous blood gas, procalcitonin, prothrombin time, partial thromboplastin time, international normalized ratio, sodium, potassium, and liver function tests were normal. Viral markers (hepatitis C antibody, hepatitis B surface antigen) were both negative. Urine and blood cultures were negative.

We immediately started enoxaparin 80 mg every 12 h. She received oral furosemide 20 mg every 12 h for 10 days. Fluid management was performed with fluid intake and output monitoring and daily laboratory tests. No significant fluid and electrolyte disturbance happened during the hospitalization.

Second TTE (5 days after the first one) showed no clot. The second chest X‐ray and pleural cavity US revealed no increase in the amount of pleural effusion. Hence, we did not perform another thoracentesis. After 2 weeks of hospitalization, she was discharged in good general condition. One year follow‐up revealed that she did not visit the IVF clinic for further treatments.

## 3 DISCUSSION

10%–15% of couples are struggling with infertility.^9^ 40% of all causes of infertility in women are related to anovulation.^1^ Medicine has provided other choices to infertile couples, but IVF still plays an important role, and its usage is still increasing.^2^

IVF has certain risks, and OHSS is the most dangerous. It is the result of overreaction of the ovary to stimulation, but the exact pathogenesis is yet to be discovered.^1^

To predict the development of OHSS, we can pay attention to risk factors: young age, low body mass index, polycystic ovarian syndrome, and previous history of OHSS are primary risk factors; the secondary risk factors depend on the ovarian response to stimulation and consist of a large number of growing follicles on the day of triggering (>14 strands with a diameter of 11 mm), a large number of oocytes retrieved and a rapid rise in estradiol levels and serum estradiol concentrations (>2500 pg/mL).^3^

Pregnancy leads to—a four to fivefold—increased risk of thromboembolic complications, including DVT and PTE.^7^ IVF and OHSS can make this hazard even more probable.^10^ Rova et al.^8^ studied all IVF deliveries for 10 years (1999–2008) and found 100‐fold increased risk of thromboembolism with OHSS compared to a fivefold increased risk without it. A systematic review performed by Sennström et al.^2^ reported doubled risk of venous thromboembolism (VTE) in the IVF group and a very high risk of VTE after OHSS. Chan et al.^11^ worked on the timing of the upper extremity thromboembolic events after IVF; they reported that the interval between embryo transfer to VTE was shorter (mean 18 days, range 3–49 days) in the OHSS group than in the group without OHSS (mean 57 days, range 14–105 days). Prescription of low‐molecular‐weight heparin to IVF patients with OHSS during the first trimester is encouraged in a systematic review performed by Sennström et al.^2^ However, they also warned that the incidence of thromboembolic events was still higher than expected in an average pregnant population.

OHSS‐related thromboses can engage both arterial and venous systems, which can lead to hazardous events: pulmonary thromboembolism,^4^ myocardial infarction,^12^ and cerebrovascular accident.^5^

Intracardiac thrombosis is another potentially life‐threatening complication. We have summarized all the reports of intracardiac thrombus in Table 1. To the best of our knowledge, this is the first reported case with a thrombus in the right atrium, possibly this intracardiac thrombosis can be the origin of the PTE. Physicians, especially obstetricians and internists, should be aware of these cardiac complications and keep OHSS in mind when encountering a patient with history of ovulation induction.

**TABLE 1:** Summary of reports of OHSS‐related intracardiac thromboses.

Author	Age/parity	Medication	Clinical manifestations	Paraclinical studies	Treatment	Outcome
Worrel et al.^13^	34 years/0	GnRH agonist/rFSH/hCG/P4	Aphasia, hemiparesis, ascites	Laboratory tests: high HGB, HCT, WBC, P4, E2; low: Protein S activity/Brain MRI: large left middle cerebral artery territory stroke and occlusion of the distal internal carotid artery/Echocardiography: Left ventricle thrombus	rtPA/AC	Resolve of intracardiac thrombus/moderate residual right spastic hemiparesis/mild difficulty with word finding
Andrejevic et al.^14^	28 years/3	Clomiphene/hMG/hCG	Obesity, hirsutism	Laboratory tests: high: ESR, positive anti‐lamin antibodies, C3, C4, IgM, IgG, CRP, RF, IgM aCL, IgM anti‐beta2GPI/Echocardiography: Left atrium thrombus	Surgery	No recurrence of throbus/persistent positive anti‐lamin antibodies, IgM aCL, IgM anti‐beta2GPI. LA activity became positive 4 months after surgery
Zamirian et al.^6^	22 years/0	Clomiphene/hCG	Dyspnea, abdominal protrusion, abdominal pain, hypotension, tachycardia, bilateral reduced breath sounds, tender abdomen, lower extremity edema	Laboratory tests: high WBC; low: HGB, HCT/Chest x‐ray: bilateral plural effusion/Abdominopelvic ultrasound: bilateral enlarged multi‐cystic ovaries and massive ascites/Echocardiography: Right ventricle thrombus	AC	Resolve of intracardiac thrombus

Abbreviations: AC, anticoagulant; aCL, anticardiolipin; anti‐beta2GPI, anti‐beta2 glycoprotein I antibodies; C3, Complement component 3; C4, Complement component 4; E2, estradiol; ESR, erythrocyte sedimentation rate; GnRH, gonadotropin‐releasing hormone; hCG, human chorionic gonadotropin; HCT, hematocrit; HGB, hemoglobin; hMG, human menopausal gonadotropin; IgG, Immunoglobulin G; IgM, Immunoglobulin M; LA, lupus anticoagulant; MRI, magnetic resonance imaging; P4, progesterone; rFSH, recombinant follicle stimulating hormone; rtPA, recombinant tissue plasminogen activator; WBC, white blood cells.

## AUTHOR CONTRIBUTIONS

**Amin Sayyadi:** Software; visualization; writing – original draft; writing – review and editing. **Mahsa Mahdavi:** Data curation; investigation. **Behnam Dalfardi:** Supervision; writing – review and editing. **Fatemeh Karami Robati:** Investigation. **Mohsen Shafiepour:** Conceptualization; data curation; supervision; writing – review and editing.

## FUNDING INFORMATION

This research did not receive any specific grant from funding agencies in the public, commercial, or not‐for‐profit sectors.

## CONFLICT OF INTEREST STATEMENT

The authors have no conflict of interest.

## ETHICAL APPROVAL

An Informed consent was received from the patient before starting the work. The study was approved by Ethics Committee of Kerman University of Medical Sciences, Kerman, Iran (Code: IR.KMU.AH.REC.1400.215).

## PATIENT CONSENT STATEMENT

Written consent was obtained from the patient regarding publishing this case report in accordance with the journal's patient consent policy.

## Supporting information


Video S1

## Data Availability

We have put the available data in this article, but in case of any questions you can directly contact the corresponding author.
